# *Morinda officinalis* oligosaccharides mitigate depression-like behaviors in hypertension rats by regulating Mfn2-mediated mitophagy

**DOI:** 10.1186/s12974-023-02715-y

**Published:** 2023-02-10

**Authors:** Lixuan Yang, Yutian Ao, Yannan Li, Baoan Dai, Jingchun Li, Wenzhe Duan, Wei Gao, Zhonghui Zhao, Zhenyun Han, Rongjuan Guo

**Affiliations:** 1 Second Clinical Medical College, Beijing University of Chinese Medicine, Beijing, 100029 People’s Republic of China; 2 Department of Clinical Psychology, Yuquan Hospital of Tsinghua University, Beijing, 100049 People’s Republic of China; 3 Department of Neurology, Shenzhen Hospital of Beijing University of Chinese Medicine, Shenzhen, 518110 Guangdong People’s Republic of China; 4 Department of Neurology, Dongfang Hospital, Beijing University of Chinese Medicine, No.6, Fangxingyuan 1st Block, Fengtai District, Beijing, 100078 People’s Republic of China; 5 Department of Traditional Chinese Medicice, Qilu Hospital of Shandong University, Jinan, 250012 Shandong People’s Republic of China

**Keywords:** Hypertension, Depression, *Morinda officinalis* oligosaccharides, Mitochondrial damage, Astrocytes

## Abstract

**Objective:**

Patients with hypertension have a risk of depression. *Morinda officinalis* oligosaccharides (MOOs) have anti-depressant properties. In this study, we aimed to determine whether MOOs can improve the symptoms of depression in individuals with hypertension.

**Methods:**

Dahl salt-sensitive rats fed with a high-salt diet were stimulated by chronic unpredictable mild stress to mimic hypertension with depression. Primary astrocytes and neurons were isolated from these rats. Astrocytes underwent LPS stimulation to simulate the inflammatory astrocytes during depression. MOOs were administrated at 0.1 mg/g/day in vivo and 1.25, 2.5, and 5 mg/mL in vitro. Mitophagy was inhibited using 5 mM 3-methyladenine (3-MA). Astrocyte-mediated neurotoxicity was detected by co-culturing astrocytes and neurons.

**Results:**

MOOs decreased systolic pressure, diastolic pressure, and mean arterial pressure, thereby improving depression-like behavior, including behavioral despair, lack of enthusiasm, and loss of pleasure during hypertension with depression. Furthermore, MOOs inhibited inflammation, astrocytic dysfunction, and mitochondrial damage in the brain. Then, MOOs promoted autophagosome and lysosome enriched in mitochondria in LPS-stimulated astrocytes. MOOs suppressed mitochondrial damage and the release of tumor necrosis factor-α (TNF-α), interleukin (IL)-6, and IL-1β in astrocytes undergoing LPS stimulation. Importantly, MOOs rescued the impaired neurons co-cultured with astrocytes. The effects of MOOs on LPS-stimulated astrocytes were reversed by 3-MA. Finally, MOOs upregulated LPS-downregulated Mfn2 expression in astrocytes. Mfn2 inhibition partly reversed the effects of MOOs on hypertension with depression. Intriguingly, Mfn2 suppression activated PI3K/Akt/mTOR pathway during MOOs treatment.

**Conclusions:**

Astrocytes develop neuroinflammation in response to mitochondrial damage during hypertension with depression. MOOs upregulated Mfn2 expression to activate the PI3K/Akt/mTOR pathway-mediated mitophagy, thereby removing impaired mitochondria in astrocytes.

**Highlights:**

1. MOOs have anti-hypertensive and anti-depressive properties.
2. MOOs inhibit inflammation and injury in astrocytes during hypertension with depression.
3. MOOs induce mitophagy activation in inflammatory astrocytes with mitochondrial damage.
4. MOOs upregulate Mfn2 expression in astrocytes.
5. Mfn2 activates mitophagy to resist mitochondrial damage in astrocytes.

## Background

Hypertension is a common cause of cardiovascular diseases such as stroke, myocardial infarction, and heart failure. Hypertension occurred in 31.1% of adults globally in 2010, and the incidence of hypertension will increase to one-third of the global population by 2025 [1, 2]. During disease progression, depression is a common complication associated with the mortality of hypertension [3]. Depression affects 53% of patients with hypertension and increases the risk of death in these individuals [4, 5]. Depressive episodes can increase the risk of suicidal ideation and behavior [6, 7]. Although pharmacological, physical, and psychological therapies have advanced, the prevention and treatment of depression are still challenging owing to heterogeneous pathogenesis, sexual dimorphism, and age specificity. Elucidating the potential mechanism underlying depressive episodes is instrumental for the development of the anti-depressive drug, which can contribute to the therapy and prognosis of hypertension with depression.

Depression is a precursor of progressive degenerative neurological disease. Depression-like conditions are related to the activation, hypofunction, and cell density of glia in the central nervous system (CNS) [8]. During depression, white matter consisting of oligodendrocytes is significantly decreased and reduced in size. Microglia are activated to release pro-inflammatory cytokines and metabolites in response to an altered neuronal microenvironment. Astrocytic dysfunction leads to changes in the emotional circuit, energy metabolism, and synaptic plasticity [9–11]. Glia-induced neuroinflammation leads to reactive oxygen species (ROS) accumulation in mitochondria, thereby resulting in oxidative stress, inflammasome enrichment, and pro-inflammatory cytokine production [12]. Astrocytes are the most abundant glial in CNS, which indicates the potential of the regulator in depression progression. Mitochondrial damage can cause the production and accumulation of ROS in astrocytes, thereby inducing the release of pro-inflammatory cytokines from astrocytes to the neuronal microenvironment. Thus, mitochondrial damage in astrocytes plays a core role in the toxicity and death of neurons during depression. To inhibit mitochondrial dysfunction, cells induce the formation of autophagosomes that are recruited into impaired mitochondria, which is called mitochondrial autophagy (mitophagy) [13]. Mitophagy plays a crucial role in the quality control of mitochondria and contributes to the removal of impaired mitochondria. Therefore, targeting mitophagy can be a research focus for the therapy of depression.

Mitofusion 2 (Mfn2) is a type of GTPase located in the outer membrane of mitochondria. It mediates the maintenance and function of mitochondria by modulating mitochondrial clustering and fusion [14, 15]. Mfn2 plays a distinctive role in the function and structure of mitochondria. The adaptive response of mitochondria to stress conditions, such as ROS production, depends on Mfn2. The deficiency of Mfn2 increases mitochondrial ROS (mtROS) and subsequently contributes to the malfunctions in the bioenergetics, functions, and metabolism of mitochondria [16, 17]. Owing to the disorder of mitochondrial fusion, Mfn2 dysfunction releases mitochondrial cytochrome C (Cyt-C) into the cytoplasm, which induces mitochondria-dependent apoptosis in neurons [15, 18]. Therefore, Mfn2 can be a potential target to improve glia-induced mitochondrial dysfunction during depression progression.

*Morinda officinalis* oligosaccharides (MOOs) are a natural extract from the root of *M. officinalis*. MOOs have shown anti-depressant properties during the progression of hypertension. MOOs mediates anti-depressant activity via glycometabolism-mediated synaptic function [19], NLRP3 inflammasomes [20], and gut–brain axis [21]. At the molecular level, MOOs play a role in the activation of tryptophan hydroxylase and the inactivation of 5-hydroxytryptophan decarboxylase in the gut, which leads to the synthesis and enrichment of cerebral 5-HT. The deficiency of cerebral 5-HT functions is the significant cause of depression [22]. Thus, MOOs can serve as a novel anti-depressive drug to modulate metabolism, cell death, and serotonin production during depression. In this study, we aimed to investigate the anti-depressive role of MOOs in hypertension.

We constructed a model of hypertension with depression in vivo and in vitro to support the hypothesis that impaired astrocyte contributes to neuroinflammation-mediated depression progression during hypertension. MOOs activated the PI3K/Akt/mTOR pathway-mediated mitophagy via Mfn2 upregulation, thereby protecting astrocytes from mitochondrial damage.

## Methods

### Animal experiments

Dahl salt-sensitive rats (Beijing Vital River Laboratory Animal Technology Co., Ltd, Beijing, China) of 6 weeks of age were used in our study to mimic hypertension with depression in vivo. The weight of rats ranged from 180 to 200 g. Before performing the experiments, the rats were fed with AIN76A normal chow diet. The rats were placed at 20 ± 1 °C under 60 ± 1% humidity with a 12-h light/dark cycle. The animal experiments were approved by the Animal Ethical and Welfare Committee (Approval No. MDKN-2022-052). Rats were randomly divided into the following 5 groups: low-salt (control; *n* = 8), high-salt (*n* = 8), high-salt + chronic unpredictable mild stress (CUMS) (*n* = 8), high-salt + CUMS + MOOs (*n* = 8), and high-salt + CUMS + MOOs + Ad-siMfn2 (*n* = 8). Rats in the low-salt group were fed with 0.12% NaCl-containing ANI76 chow for 28 days. Rats that were fed with high-salt AIN76A chow containing 4% NaCl for 28 days developed symptoms of hypertension. To mimic hypertension with depression, rats fed with a high-salt diet were stimulated by CUMS as described previously [23] with a slight modification. CUMS included food deprivation for 24 h, swimming for 5 min at 4 °C, tail clamping for 1 min, swimming for 5 min at 45 °C, level shaking for 10 min at 60 times/min, 45° of cage tilting for 24 h, and inversion of the light/dark cycle for 24 h. Rats were randomly exposed to different stressors every day. These stimulation methods were performed for 28 days, during which each stimulation was applied 4 times. For pharmacological treatment, rats were treated with MOOs (0.1 mg/g of body weight/day; Beijing Tongrentang Ltd. Co., Beijing, China) by oral gavage once daily for 28 days during CUMS procedures. Rats in non-MOOs groups were orally administrated with the vehicle equivalent to MOOs, including control, high salt and high-salt + CUMS groups. Mfn2 siRNA was synthesized and expressed in the adenovirus (Ad) vector (GenePharma, Shanghai, China). Rats were injected with Ad-siMfn2 (5 × 10^9^ PFU) on the lateral ventricle once every 7 days for 4 times. The experiment procedures were shown as follows:
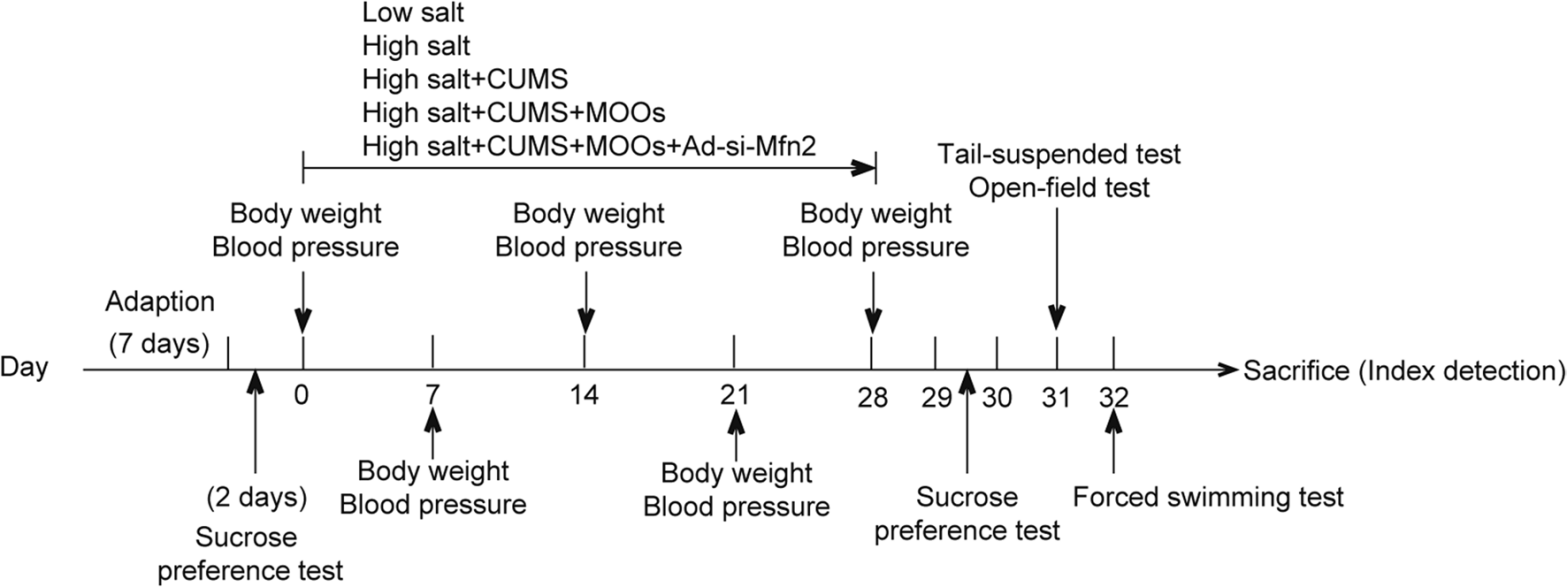


### Blood pressure monitoring

Systolic blood pressure, diastolic blood pressure, and mean arterial pressure of rats were measured using a noninvasive blood pressure monitor (ADInstruments, USA) on day 0, 7, 14, 21 and 28. The measurements were taken when rats were conscious.

### Behavior tests

#### Sucrose preference test

The food intake and the water intake before modeling were 10 g/100 g and 13 mL/100 g during 24 h, indicating the normal intake of food and water in rats. The baseline of sucrose preference test was tested 2 days before the modeling. After the 28-day modeling, sucrose preference test was performed on day 29–30. Two bottles were placed in each cage, one containing 100 mL of 1% sucrose solution (v/v) and another containing 100 mL of pure water. After habitation for 12 h, one rat was placed in each cage with free access to the two bottles for 24 h. The volume of the remaining solutions was recorded. Sucrose preference was calculated using the following formula: [(sucrose consumption)/(water consumption + sucrose consumption)] × 100%. Rats adapted to the experimental environment for 30 min before testing.

#### Tail-suspended test

On day 31, tail-suspended test was performed after 30-min adaptation. In a sound-isolated environment, each rat was suspended with adhesive tape (57 cm between the tape and platform) for 6 min. The immobility time of the rats in the last 4 min was recorded when rats were not actively struggling.

#### Open-field test

This test was processed 12 h after tail-suspended test. After 30-min adaptation, each rat was placed in the center of a white chamber box (100 × 100 × 40 cm^3^). Rats could freely explore the box for 5 min. Their movement was recorded to calculate the horizontal score and vertical score. In this study, the horizontal score was described by the square number crossed by a limb of the rat, as the vertical score was by the standing times of the hind limb of the rat. Before each test, the bottom of the box was cleaned using alcohol cotton.

#### Forced swimming test

Rats adapt to the experimental environment for 30 min before testing. On day 32, each rat was placed in a glass cylinder (diameter: 21 cm and height: 46 cm) containing 35-cm-depth water at 24 ± 1 °C. Each rat was forced to swim for 15 min. Rats were placed in rearing cages after they were dried. On day 33, rats were individually placed in the cylinder for force swimming for 6 min (pre-test of 2 min and test of 4 min). The immobility time of rats was recorded in the last 4 min.

#### Hematoxylin and eosin (H&E) staining

After the last behavior test, the rats were euthanized using 150 mg/kg pentobarbital sodium, and the brain tissues were isolated. Brain tissues embedded in paraffin were cut into sections of 5 µm thickness. Then, dewaxed sections were stained using hematoxylin for 5 min and eosin for 1 min. Finally, stained sections were dehydrated using 70%, 80%, 90%, and 100% of ethanol solution for 10 s each. Sections fixed in neutral balsam were observed under a microscope (Olympus, Japan).

### Enzyme-linked immunosorbent assay (ELISA)

Serum, tissue lysates, and cell supernatant were collected to detect the levels of tumor necrosis factor (TNF)-α, interleukin (IL)-1β, and IL-6 using ELISA. TNF-α (E-EL-R2856c, Elabscience, Wuhan, China), IL-1β (E-EL-R0012c, Elabscience), and IL-6 (E-EL-R0015c, Elabscience) kit were used. ELISA was performed according to the manufacturer’s protocol.

### Mitochondrial morphology observation

Brain tissues fixed in 2.5% glutaraldehyde and 1% osmic acid for 2 h was dehydrated using 70–100% acetone. Subsequently, tissues embedded in epoxy resin at 60 °C for 36 h were cut into 1-μm-thick sections. Sections were stained using Janus Green B (Solarbio, Beijing, China) for 1 min. Mitochondrial morphology was observed using a transmission electron microscope (JEOL, Tokyo, Japan).

### Cell culture and treatments

Primary astrocytes were isolated from the sheared brain tissues of rats undergoing mechanical dissociation at 6 r/min for 30 min in Earle’s balanced salt solution (Gibco, Carlsbad, CA, USA) supplemented with DNase I. After filtration using a 70-µm cell strainer, cells resuspended in 30% Percoll (Solarbio) were centrifuged at 300 × *g* for 30 min, followed by another centrifugation at 300 × *g* for 10 min. Cells resuspended in the astrocyte medium (ScienceCell, Carlsbad, CA, USA) were cultured in T25 flasks (Thermo Fisher Scientific, Waltham, MA, USA) supplemented with 5 mL astrocyte medium containing 2% fetal bovine serum (Gibco) and 1% astrocyte growth supplement (ScienceCell), and 1% antibiotic solution (ScienceCell) under the condition of 37 °C and 5% CO_2_. Also, primary neurons were isolated from brain tissues. Sheared tissues were incubated in the mixed solution consisting of 0.25% trypsin (Solarbio) plus 1% collagenase I (Solarbio) for 30 min at 37 °C. After filtration using a 100-µm cell strainer, cells resuspended in neuronal medium (ScienceCell) were incubated in T25 flasks containing neuronal medium with 1% neuronal growth supplement (ScienceCell) and 1% antibiotic solution (ScienceCell) at 37 °C/5% CO_2_. In non-MOOs groups, cells were incubated with the vehicle at the equal volume to MOOs.

### Experiment 1

Astrocytes were divided into the following 5 groups: (1) control group cultured in a normal medium. (2) LPS group cultured in cell medium containing 1 µg/mL LPS for 24 h. (3) LPS + MOOs (1.25 mg/mL) group pretreated with 1.25 mg/mL MOOs for 24 h and then cultured in 1 µg/mL LPS for 24 h. (4) LPS + MOOs (2.5 mg/mL) group undergoing a 24-h treatment with 2.5 mg/mL MOOs and then a 24-h LPS stimulation. (5) LPS + MOOs (5 mg/mL) group with 5 mg/mL MOOs treated cells for 24 h and a 24-h LPS stimulation. We screened the optimum concentration of MOO among the following concentrations: 1.25, 2.5, and 5 mg/mL.

### Experiment 2

Astrocytes were divided into the following 4 groups: control, LPS, LPS + MOOs, and LPS + MOOs + 3-MA. Experiments for the control and LPS groups were the same as experiment 1. The astrocytes in the LPS + MOOs group and LPS + MOOs + 3-MA group were pretreated with 5 mg/mL MOOs (in medium) for 24 h and then stimulated using 1 µg/mL LPS for 24 h. However, astrocytes in the LPS + MOOs + 3-MA group underwent 5 mM 3-methyladenine (3-MA, an inhibitor of autophagy; MedChem Express, Monmouth Junction, NJ, USA) when stimulated using LPS. Additionally, neurons were co-cultured with astrocytes in neuronal medium containing 1% neuronal growth supplement.

### Experiment 3

Mfn2 siRNA (siMfn2, sense: 5′-CCA AAU UGC UCA GGA AUA ATT-3′, antisense: 5′-UUA UUC CUG AGC AAU UUG GTT-3′; Sangon Biotech, Shanghai, China) were designed and synthesized to downregulate Mfn2 expression in astrocytes. NC siRNA (siNC, sense 5′-UUC UCC GAA CGU GUC ACG UTT3′, antisense 5′-ACG UGA CAC GUU CGG AGA ATT-3′) was used as the negative control of Mfn2 siRNA. Cell transfection was processed using a Lipofectamine 3000 kit (Invitrogen, Carlsbad, CA, USA) with 1.6 µg of siMfn2 or siNC. Mfn2 expression was determined by quantitative polymerase chain reaction (qPCR) 48 h after transfection.

Astrocytes were divided into the following 4 groups: control, LPS, LPS + MOOs + siNC, and LPS + MOOs + siMfn2. Cell transfection was performed 48 h before MOOs treatment. Administration procedures of LPS and MOOs were the same as that in Experiment 1.

### Cell Counting Kit-8 (CCK-8) assay

Cells from different groups were pre-cultured in a 96-well plate for 24 h at 37 °C under 5% CO_2_. CCK-8 reagent (Dojindo, Kumamoto, Japan) of 10 µL was added to each well, and cells were cultured for 4 h at 37 °C under 5% CO_2_. The absorbance of each well was obtained at 450 nm using a microplate reader (Thermo Fisher Scientific). Cell viability was calculated using the absorbance value.

### Flow cytometry

Apoptosis in neurons was determined with an Annexin V-FITC Apoptosis Staining Kit (Abcam, Cambridge, MA, USA) using a flow cytometer. Neurons with a density of 1 × 10^5^ from different groups were collected for centrifugation. Then, neurons resuspended in Annexin V binding buffer were incubated with 5 µL of Annexin V-FITC and 5 µL propidium iodide (PI) for 5 min at room temperature in the absence of light. The apoptosis rate of neurons was determined using flow cytometry with a FITC signal detector and phycoerythrin emission signal detector.

### ROS measurement

Intracellular and mtROS were labeled with DCFH-DA (50101ES01, YEASEN, Shanghai, China) and mitoSOX probe (M36008, Thermo Fisher Scientific), respectively. Astrocytes from different groups were seeded in 96-well plates covered with coverslips. For detecting the cytoplasmic level of ROS, astrocytes were incubated with diluted DCFH-DA solution for 30 min at 37 °C in the dark. For detecting the level of mtROS, astrocytes were incubated in 1 mL of 5 µM mitoSOX solution for 10 min at 37 °C without light. Cytoplasmic and mtROS were observed under a fluorescence microscope (Olympus, Tokyo, Japan).

### Mitochondrial membrane potential detection

Mitochondrial membrane potential was determined using a JC-1 probe (M8650, Solarbio). Astrocytes were pre-cultured in 6-well plates, and 1 mL of fresh medium was added to each well to replace the old medium. Then, 1 mL of JC-1 solution was added to each well, and the cells were incubated for 20 min at 37 °C. After the removal of the medium, 2 mL of fresh medium was added to each cell. Mitochondrial membrane potential was determined using a fluorescence microscope at Ex/Em = 490/530 nm.

### Fluorescent localization

For the detection of autophagosome and autolysosome, cells were pre-cultured in 6-well plates and transiently transfected with 20 multiplicity of infection (MOI) of GFP-mRFP-LC3 adenovirus (probe for autophagy; HB-AP210 0001, Hanbio, Shanghai, China). The fluorescent image was captured using a confocal laser scanning microscope (Olympus) 24 h after infection. To detect the localization of mitochondria, cells were incubated with 100 nM Mito-Tracker Red (C1035, Beyotime, Shanghai, China) for 30 min at 37 °C. The fluorescent organelles were observed using a confocal laser scanning microscope. Primary astrocytes and neurons were identified using a fluorescent GFAP and NSE, respectively. Autophagy was detected using a fluorescent LC3. In brief, cells fixed in 4% paraformaldehyde (Solarbio) were incubated in a block buffer containing PBS, 5% normal serum (v/v), and 0.3% Triton-100 X (v/v) for 1 h. Subsequently, cells were incubated with diluted anti-GFAP (#80,788, 1:200, Cell Signaling Technology, Danvers, MA, USA), anti-NSE (ab53025, 2 µg/mL, Abcam), anti-NeuN (ab177487, 1:200, Abcam), and anti-LC3 (ab48394, 1 µg/mL, Abcam) overnight at 4 °C, followed by incubating with diluted secondary antibody (#8889, 1:500, Cell Signaling Technology; ab150077, 1:500, Abcam) at room temperature for 1 h in dark. Cells were labeled by the DAPI probe (#8961, Cell Signaling Technology). The fluorescent images were captured using a confocal laser scanning microscope.

### qPCR assay

Total RNA was extracted from cell lysate using Trizol reagent (Solarbio), followed by its reverse transcription to cDNA and real-time quantification using a TaqMan One-Step RT-qPCR Kit (Solarbio) under ABI7000 (Applied Biosystems, Foster City, CA, USA). Relative expression of mRNA was calculated using the formula 2^–ΔΔCt^ and normalized using GAPDH as the control. Primer sequences designed and synthesized by Sangon Biotech were as follows: Drp1-forward, 5′-ACA ACA GGA GAA GAA AAT GGA GT-3′; Drp1-reverse, 5′-TCA CTT GTG GCC CAG GTA TG-3′; Fis1-forward, 5′-GAA TAC GCC TGG TGC CTG GTT C-3′; Fis1-reverse, 5′-CGT TGG GCG AGA AAA CCT TG-3′; Mfn1-forward, 5′-CGT GGC AGC AGC AGA GAA GAG-3′; Mfn1-reverse, 5′-GGT GTA CCC GCA GTG AAG AA-3′; Mfn2-forward, 5′-AGA GGC GAT TTG AGG AGT GC-3′; Mfn2-reverse, 5′-CCT CCT CCG TGA CCT CCT TGA TC-3′; OPA1-forward, 5′-ATT TCG CTC CTG ACC TGG AC-3′; OPA1-reverse, 5′-GAA GAC ATA ATC CCG CTG CTC CTC-3′.

### Western blot

Protein from tissue lysate or cell lysate obtained using RIPA lysis buffer (Beyotime) was separated by sodium dodecyl sulfate–polyacrylamide gel electrophoresis (SDS-PAGE) (Bio-Rad, Hercules, CA, USA), followed transferring to polyvinylidene difluoride (PVDF) membranes place on ice using a mini transblotting system (Bio-Rad). Membranes were incubated with the block buffer containing 5% skimmed milk for 90 min. Then, membranes were incubated with diluted primary antibody at 4 °C overnight, followed by the incubation with secondary antibody (ab205718, 1:10,000, Abcam) for 1 h at 37 °C. Blots were visualized using an ECL kit (Abcam). A gel imaging system (Bio-Rad) was used to capture and analyze the blots. GAPDH was used as the loading control of proteins. Primary antibodies included anti-Mfn2 (ab124773, 1:1000, Abcam), anti-LC3 (ab192890, 1:2000, Abcam), anti-p62 (ab109012, 1:10,000, Abcam), anti-Parkin (#32,833, 1:1000, Cell Signaling Technology), anti-TOMM20 (ab186735, 1:2000, Abcam), anti-COX IV (ab202554, 1:2000, Abcam), anti-cleaved caspase-3 (#9661, 1:1000, Cell Signaling Technology), anti-cleaved caspase-9 (AF5240, 1:1000, Affinity Biosciences, Liyang, China), anti-PI3K (#4257, 1:1000, Cell Signaling Technology), anti-p-PI3K (AF3242, 1:1000, Affinity Biosciences), anti-Akt (#4685, 1:1000, Cell Signaling Technology), anti-p-Akt (#4060, 1:2000, Cell Signaling Technology), anti-mTOR (#2983, 1:1000, Cell Signaling Technology), anti-p-mTOR (#5536, 1:1000, Cell Signaling Technology), and anti-GPADH (#5174, 1:1000, Cell Signaling Technology).

### Statistical analysis

Data are shown as the mean ± standard deviation. All experiments were performed in triplicates. For comparison among the multiple groups, the one-way analysis of variance test was performed, followed by the post hoc comparison of Tukey’s multiple comparison test. Student’s *t* test was used to determine statistical differences between two groups. A statistical difference when *p* was < 0.05 at a 95% confidence interval was considered significant.

## Results

### MOOs relieved depression-like behaviors of the hypertensive rats

**Fig. 1:**
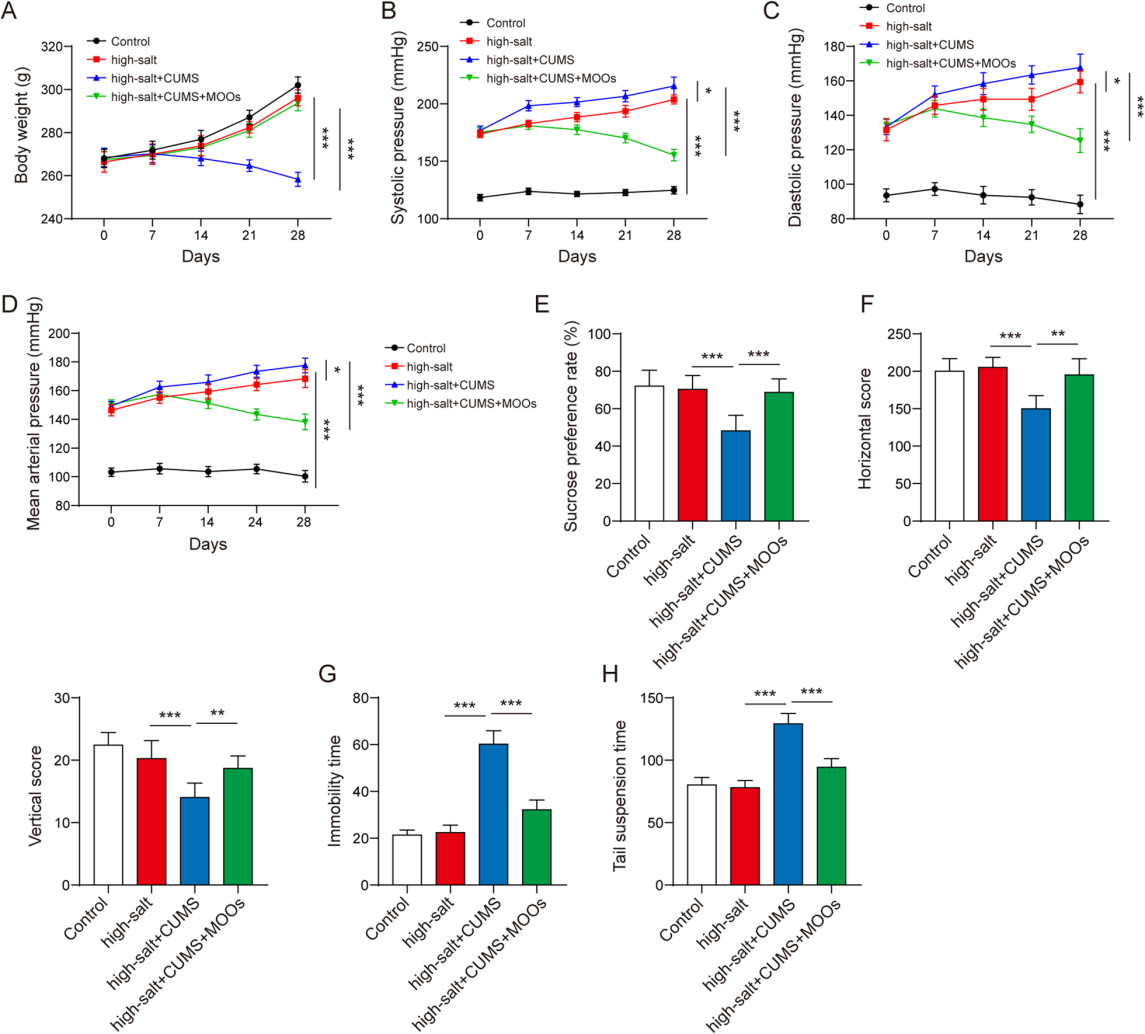
*Morinda officinalis* oligosaccharides relieved depression-like behaviors in hypertensive rats. **A** Monitoring the body weight of the rats. The systolic pressure (**B**), diastolic pressure (**C**), and the mean arterial pressure (**D**) of the rats were determined. Sucrose preference test (**E**), open-field test (**F**), forced swimming test (**G**), and tail suspension test (**H**) of the indicated rats (*n* = 8 rats/group). Data are shown as the mean ± standard deviation. *p < 0.05, **p < 0.01, and ***p < 0.001 by one-way analysis of variance followed by Tukey’s post hoc test

First, we determined the therapeutic role of MOOs in hypertension symptoms during depressive stimulation. CUMS significantly decreased the body weights of the hypertensive rats (Fig. 1A). However, MOO treatment increased the CUMS-decreased body weights of the rats during hypertension progression (Fig. 1A). CUMS increased the hypertension-induced rise in systolic and diastolic pressures in the rats; MOOs decreased systolic and diastolic pressures of the hypertensive rats with CUMS (Fig. 1B, C). Similarly, the mean arterial pressure was reduced by MOOs in the depressive hypertensive rats (Fig. 1D). Then, we measured changes in depression-like behaviors of the hypertensive rats with CUMS. Hypertension made no difference in depression-like behaviors of the rats according to the results of the sucrose preference test (Fig. 1E), open-field test (Fig. 1F), forced swimming test (Fig. 1G), and tail test (Fig. 1H). The hypertensive rats experiencing CUMS were characterized by behavioral despair, lack of enthusiasm, and pleasure loss (Fig. 1E-H). Importantly, MOOs significantly improved these depression-like behaviors caused by CUMS (Fig. 1E-H).

### The protective effect of MOOs on brain injury in depressive hypertensive rats

**Fig. 2:**
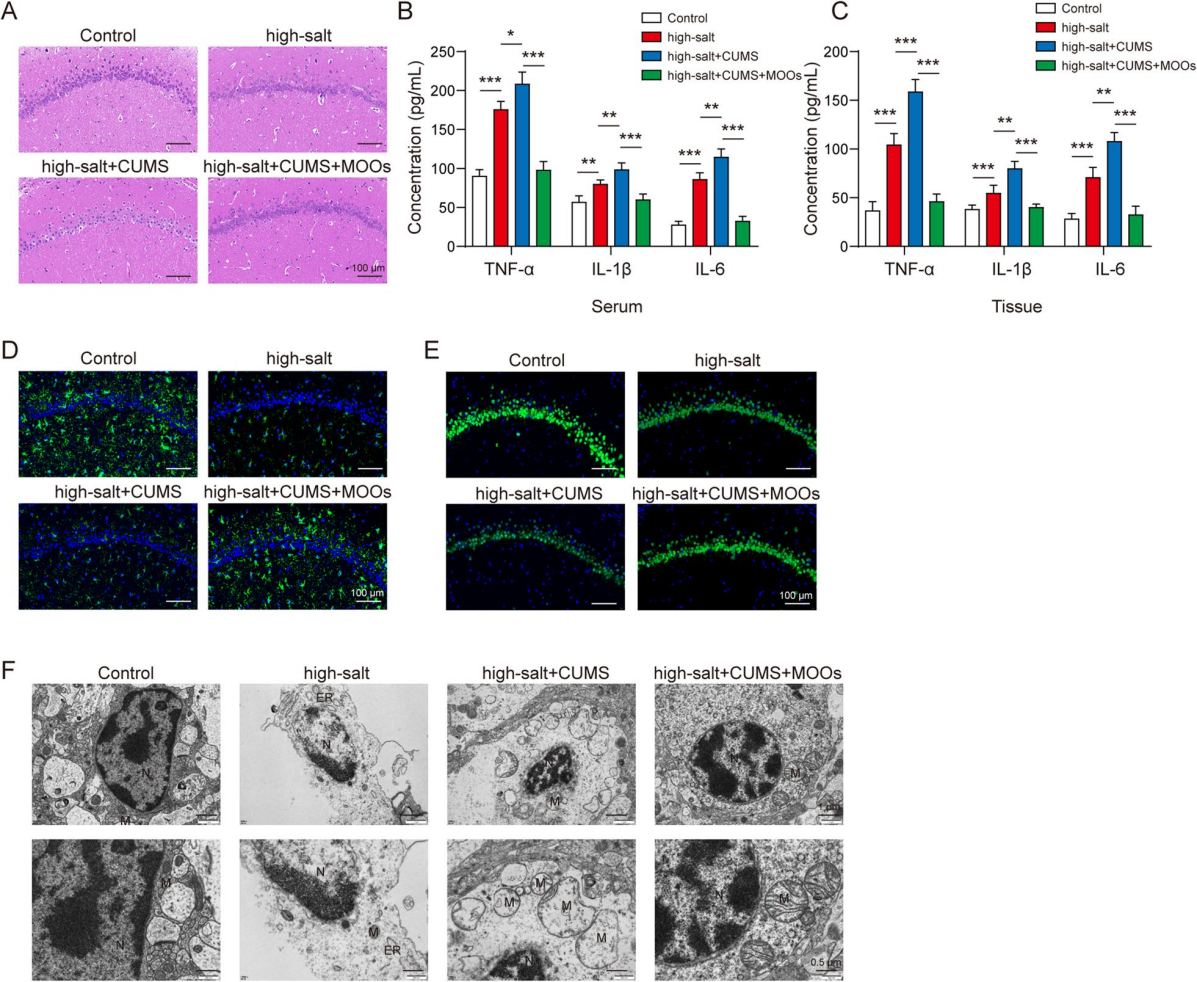
Protective effect of *Morinda officinalis* oligosaccharides on brain injury in depressive and hypertensive rats. **A** Histological changes in brain tissues were observed by hematoxylin and eosin staining. Tumor necrosis factor α, interleukin (IL)-1β, and IL-6 levels in serum (**B**) and brain tissues (**C**) were determined by enzyme-linked immunosorbent assay. **D** Immunocytochemistry of glial fibrillary acidic protein in astrocytes. **E** Death of neurons was tested and pictured by immunostaining for neuronal nuclear protein (green). **F** Mitochondrial morphology was observed using a transmission electron microscope (*n* = 8 rats/group). Data are shown as the mean ± standard deviation. *p < 0.05, **p < 0.01, and ***p < 0.001 by one-way analysis of variance followed by Tukey’s post hoc test

First, we observed a protective role of MOOs in cerebral histopathology during depression and hypertension. In the brain of the healthy rats, glial cells and neurons grew normally showing the clear structure of nuclear membranes and the nucleolus (Fig. 2A). CUMS increased hypertension-induced karyolysis, inflammatory infiltration, swollen mitochondria, pyknotic nuclei, and neurons with a blurred structure in the cerebral tissues of the rats, which were improved by MOO treatment (Fig. 2A). Then, we found that MOOs inhibited inflammation in the hypertensive rats with CUMS. Hypertension induced the increase in TNF-α, IL-1β, and IL-6 levels in the brain and serum, and CUMS further increased the levels of these pro-inflammatory cytokines (Fig. 2B, C). Importantly, MOO treatment alleviated inflammation in the brain and serum (Fig. 2B, C). Based on GFAP (a marker of astrocytes) and NeuN (a marker of neurons) levels, we found a decrease in active astrocytes (Fig. 2D) and an increase in dead neurons (Fig. 2E) in the depressive hypertensive rats. However, MOOs promoted the activation of astrocytes and inhibited the death of neurons during hypertension with depression (Fig. 2D, E). Although hypertension and depression induced mitochondrial damage such as swollen mitochondria, adventitia rupture, cavitation, and disrupted cristae in the rat brains (Fig. 2F), MOOs played a protective role in maintaining mitochondrial integrity (Fig. 2F).

### MOOs inhibited inflammatory factor release and mitochondrial damage in astrocytes

**Fig. 3:**
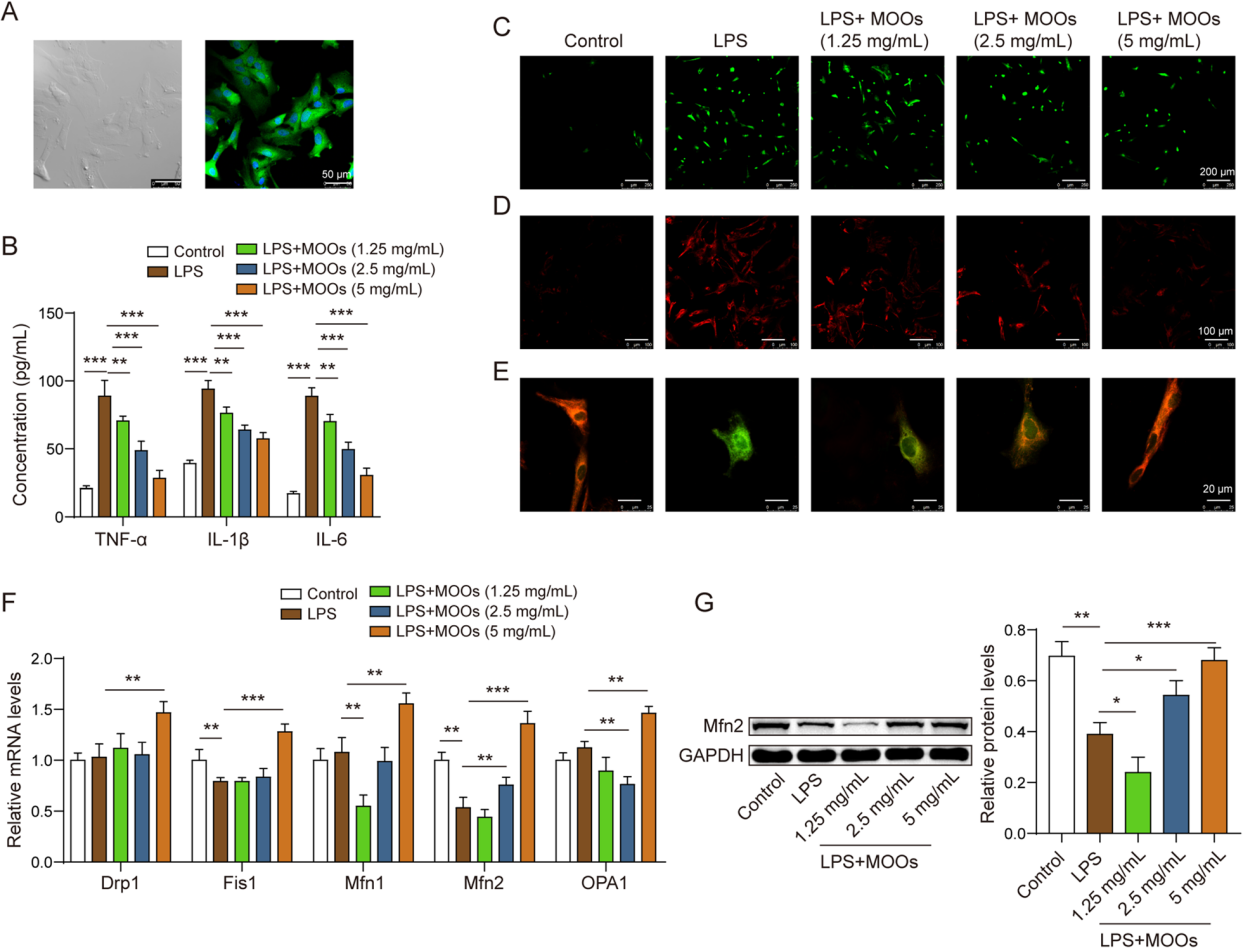
*Morinda officinalis* oligosaccharides inhibited inflammatory factor release and mitochondrial damage in astrocytes. **A** Astrocytic morphology was identified by light microscopy and fluorescent staining for glial fibrillary acidic protein. **B** Enzyme-linked immunosorbent assay for tumor necrosis factor α, interleukin (IL)-1β, and IL-6 levels in astrocyte supernatants. Intracellular (**C**) and mitochondrial (**D**) reactive oxygen species levels were assessed using the DCFDA probe and MitoSOX probe. **E** Mitochondrial membrane potential was determined using the JC-1 probe. **F** Screening of differentially expressed mitochondrial kinetic molecules by quantitative polymerase chain reaction. **G** Relative expression of Mfn2 was determined by western blotting. Data are shown as the mean ± standard deviation of three independent experiments. *p < 0.05, **p < 0.01, and ***p < 0.001 by one-way analysis of variance followed by Tukey’s post hoc test

To determine the astrocyte-mediated mechanism of MOOs in hypertension with depression, primary astrocytes from the cerebral tissues of the healthy rats were cultured in vitro (Fig. 3A). These astrocytes were stimulated with LPS to mimic the inflammatory condition during hypertension with depression. MOOs significantly repressed the LPS-induced increase in cytokines in the astrocytes in a concentration-dependent manner (Fig. 3B). Similarly, oxidative stress was altered in the LPS-stimulated astrocytes. We found that LPS increased intracellular (Fig. 3C) and mitochondrial (Fig. 3D) ROS levels in the astrocytes, which were inhibited by MOO treatment in a concentration-dependent manner. Furthermore, we visualized mitochondrial membrane potential in the astrocytes using the JC-1 probe to determine mitochondrial dysfunction. MOOs increased mitochondrial membrane potential in the LPS-stimulated astrocytes in a concentration-dependent manner (Fig. 3E). We measured the expressions of the genes of mitochondrial kinetic molecules, including Drp1, Fis1, Mfn1, Mfn2, and OPA1, in the astrocytes during LPS stimulation. Results showed that only Mfn2 expression was affected by both LPS stimulation and MOO treatment (Fig. 3F), which was selected for further study. Moreover, MOOs at 2.5 and 5 mg/mL upregulated the LPS-downregulated Mfn2 expression in the astrocytes (Fig. 3G). However, Mfn2 was increased because of MOOs treatment at 2.5 and 5 mg/mL (Fig. 3G).

### MOOs inhibited neurotoxicity in astrocytes by enhancing autophagy

**Fig. 4:**
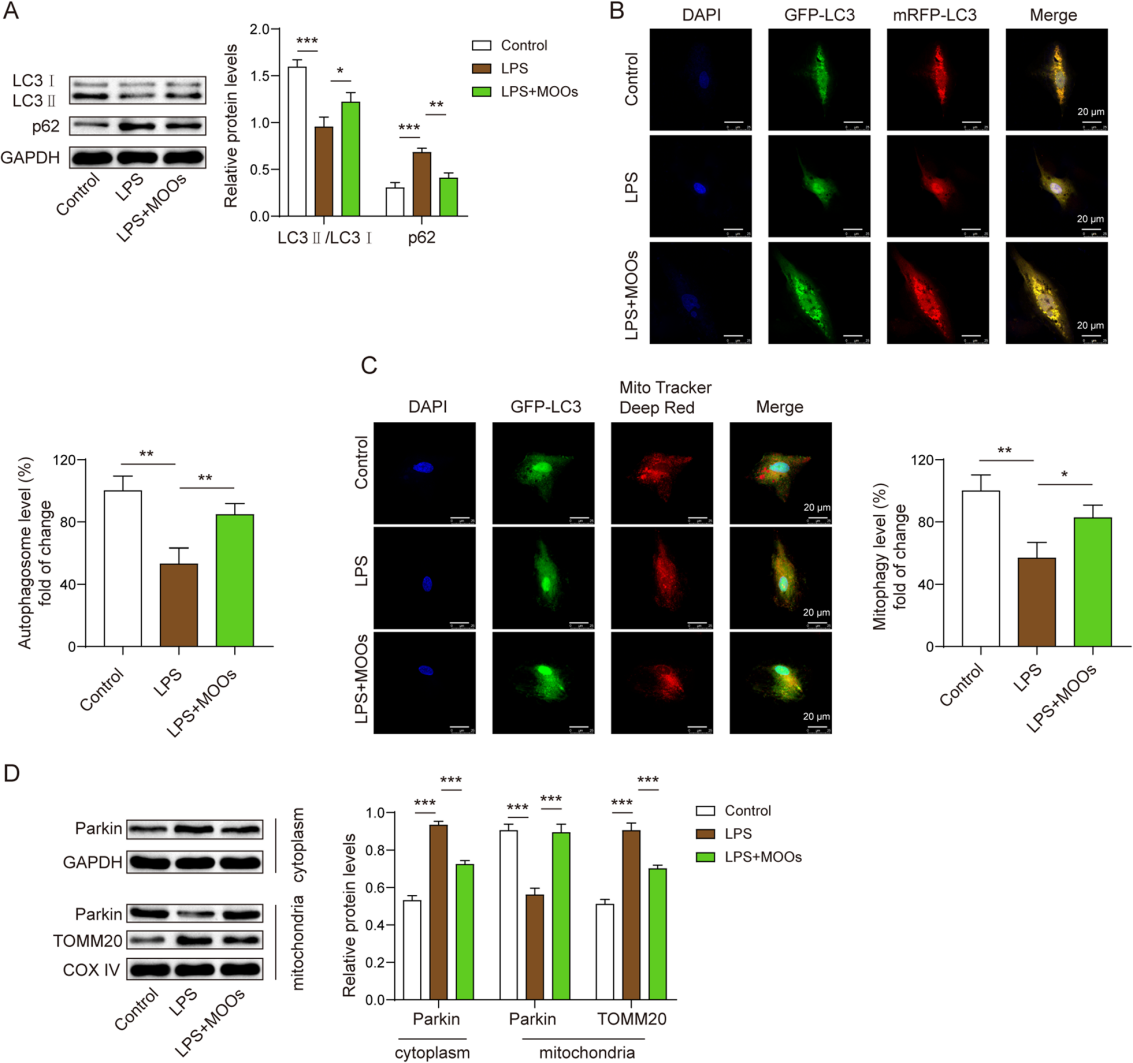
*Morinda officinalis* oligosaccharides promoted autophagic flux and mitophagy in astrocytes. **A** Levels of LC3 and p62 were determined by western blotting. **B** Fluorescent localization of the autophagosome (yellow) and autolysosome (red) using the GFP-mRFP-LC3 probe. **C** Fluorescent localization of the autophagosome and mitochondria using the GFP-LC3 probe and Mito Tracker Deep Red probe, respectively. **D** Levels of mitophagy-associated proteins, including Parkin and TOMM20, in the cytoplasm and mitochondria were determined by western blotting. Data are shown as the mean ± standard deviation of three independent experiments. *p < 0.05, **p < 0.01, and ***p < 0.001 by one-way analysis of variance followed by Tukey’s post hoc test

We found that MOO treatment promoted autophagic flux and mitophagy in the LPS-stimulated astrocytes. LPS stimulation decreased LC3 levels and increased p62 levels in the astrocytes, indicating that astrocytic autophagy was inhibited during LPS stimulation (Fig. 4A). However, MOO treatment reversed the alterations in LC3 and p62 (Fig. 4A). We further found that the autophagosomes and autolysosomes were repressed in the cytoplasm during LPS stimulation (Fig. 4B). However, MOOs activated autophagosome and autolysosome formation in the cytoplasm (Fig. 4B) and promoted autophagosome enrichment in the mitochondria of the LPS-stimulated astrocytes (Fig. 4C). At the molecular level, cytoplasmic Parkin failed to transfer to the mitochondria, resulting in the stability of TOMM20 (Fig. 4D). However, MOOs contributed to transferring Parkin to the mitochondria, leading to TOMM20 degradation (Fig. 4D).

**Fig. 5:**
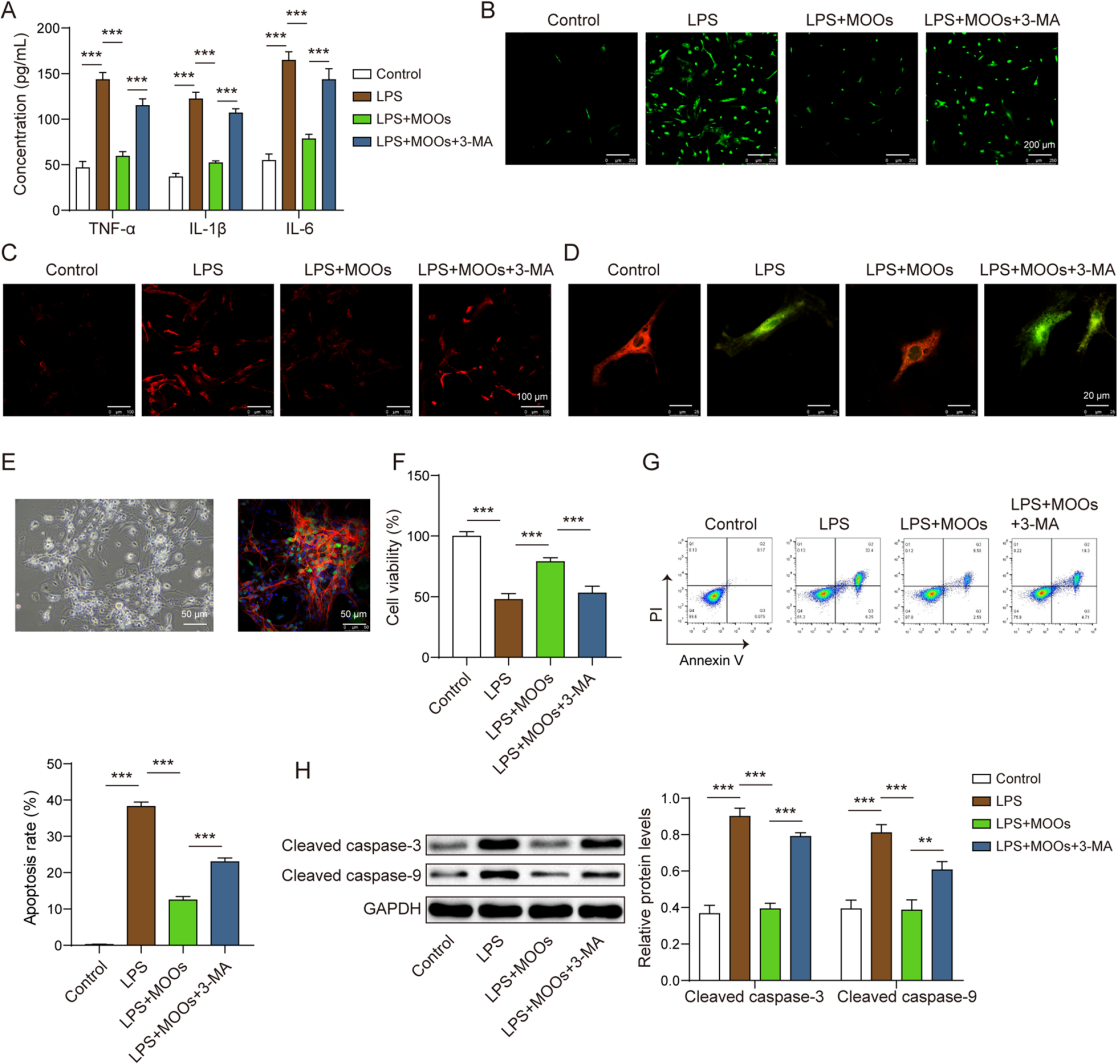
Autophagy inhibitors aggravated neuronal toxicity inhibited by *Morinda officinalis* oligosaccharides by preventing mitochondrial damage. **A** Tumor necrosis factor α, interleukin (IL)-6, and IL-1β levels in astrocyte supernatants were determined by enzyme-linked immunosorbent assay. Intracellular (**B**) and mitochondrial (**C**) reactive oxygen species levels were assessed using the DCFDA probe and MitoSOX probe. **D** Mitochondrial membrane potential was assessed using the JC-1 probe. **E** Isolation and identification of primary neurons by light microscopy and fluorescent staining for neuron-specific enolase. **F** Cell viability of neurons was determined by cell counting kit-8 assay. **G** Apoptosis of neurons was determined by flow cytometry. **H** Apoptosis-related protein levels (cleaved caspase-3 and cleaved caspase-9) in neurons were determined by western blotting. Data are shown as the mean ± standard deviation of three independent experiments. **p < 0.01, and ***p < 0.001 by one-way analysis of variance followed by Tukey’s post hoc test

We further determined whether MOOs inhibited astrocytic neurotoxicity by enhancing autophagy. As mentioned earlier, MOOs inhibited inflammation, ROS accumulation, and mitochondrial damage in the LPS-stimulated astrocytes. To determine whether autophagy mediated the protective role of MOOs in these astrocytes, 3-MA was used to inhibit astrocytic autophagy during MOO treatment. In the MOO-treated astrocytes, 3-MA increased pro-inflammatory cytokine levels (Fig. 5A) as well as intracellular and mtROS levels (Fig. 5B, C) and reduced mitochondrial membrane potential (Fig. 5D). Primary neurons (Fig. 5E) from the rats were cultured to determine the role of the autophagy–astrocyte–neuron axis during MOO treatment. The neurons were treated with conditioned media from astrocytes exposed to LPS and MOOs with or without 3-MA. We found that MOOs increased cell viability (Fig. 5F) and decreased apoptosis in these neurons (Fig. 5G) with the downregulated expression of cleaved caspase-3 and 9 genes (Fig. 5H). The effects of MOOs on cell viability and apoptosis in these neurons were reversed by 3-MA (Fig. 5F–H).

### Mfn2 mediated MOO-associated mitophagy via the PI3K/Akt/mTOR pathway and neuroprotection

**Fig. 6:**
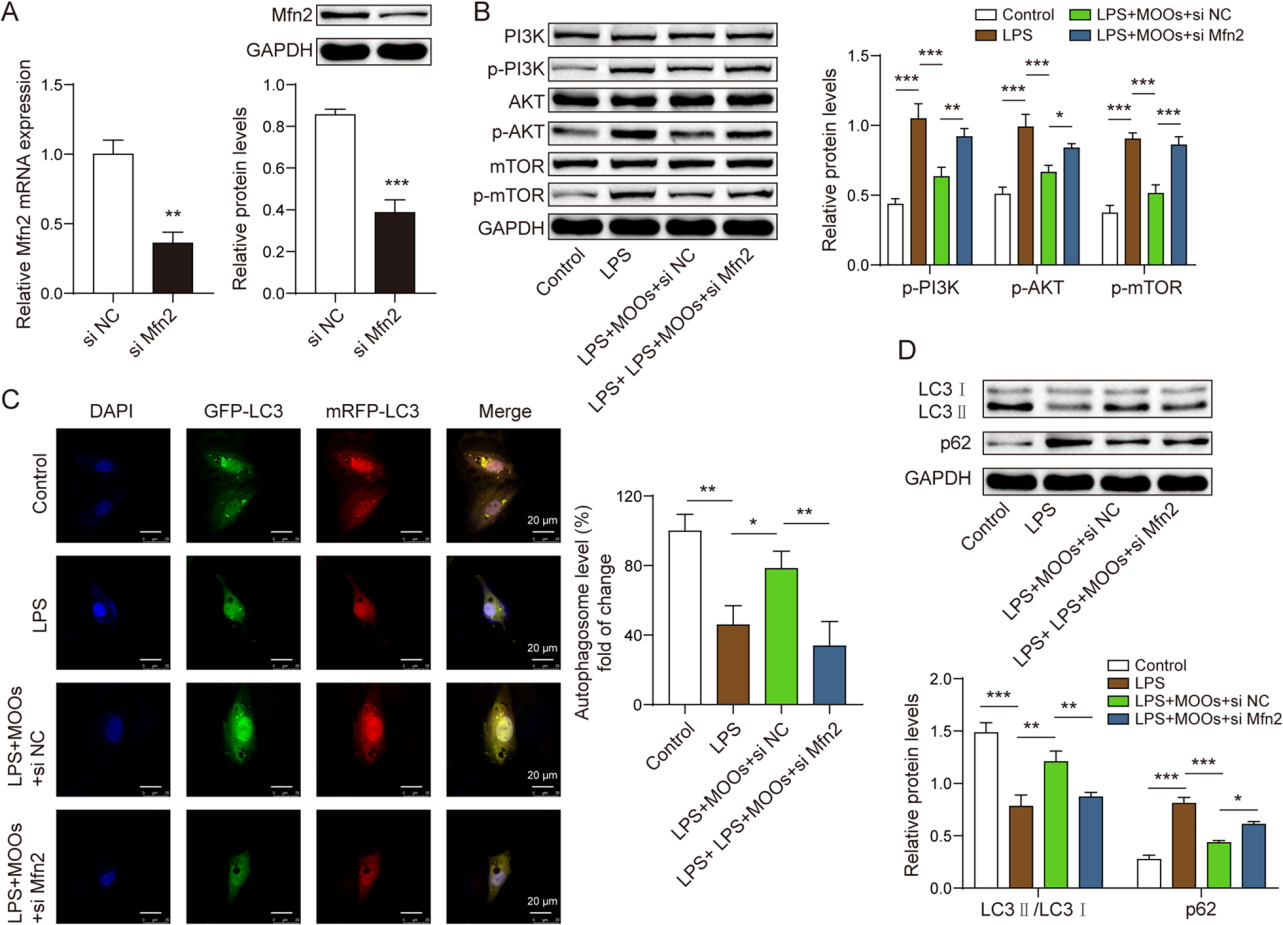
Mfn2 mediated inactivation of the phosphoinositide 3-kinase (PI3K)/AKT/mammalian target of rapamycin (mTOR) involved in the pro-autophagic effect of *Morinda officinalis* oligosaccharides. **A** Mfn2 expression was determined by quantitative polymerase chain reaction (qPCR) and western blotting. **B** The PI3K/Akt/mTOR pathway-related protein levels were determined by western blotting. **C** Fluorescent localization of the autophagosome (yellow) and autolysosome (red) using the GFP-mRFP-LC3 probe. **D** Autophagy-related protein levels, including LC3 and p62, were determined by western blotting. Data are shown as the mean ± standard deviation of three independent experiments. *p < 0.05, **p < 0.01, and ***p < 0.001 by Student’s *t* test or one-way analysis of variance followed by Tukey’s post hoc test

**Fig. 7:**
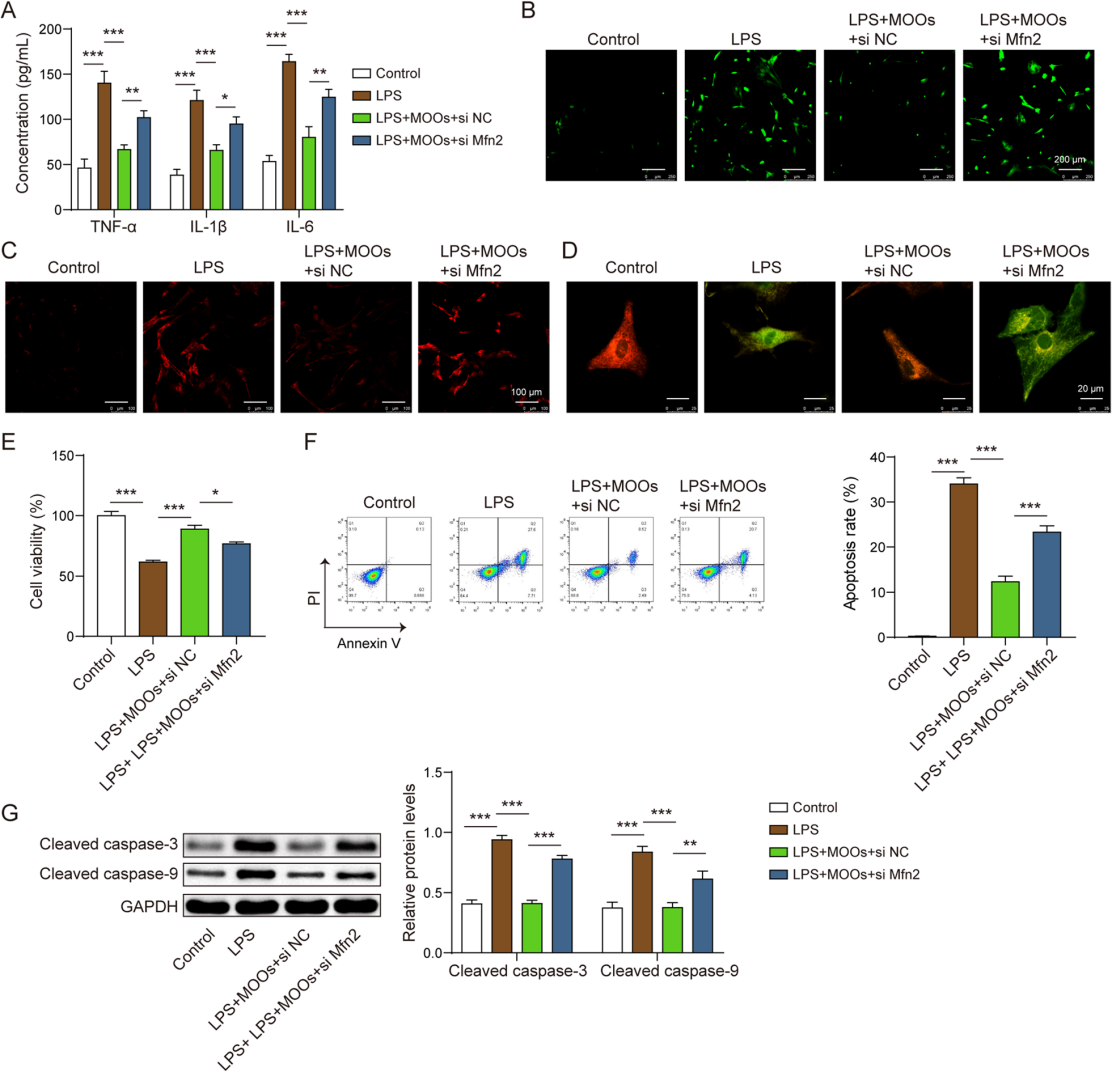
Mfn2 knockdown abolished the protection of *Morinda officinalis* oligosaccharides in neuronal toxicity. **A** Tumor necrosis factor α, interleukin (IL)-6, and IL-1β levels in astrocyte supernatants were determined by enzyme-linked immunosorbent. **B** Intracellular reactive oxygen species (ROS) levels were determined using the DCFDA probe. **C** Mitochondrial ROS levels were determined using the MitoSOX probe. **D** The JC-1 probe for mitochondrial membrane potential detection. **E** Cell counting kit-8 assay for cell viability measurement. **F** Apoptosis of neurons was determined by flow cytometry. **G** Apoptosis-related protein levels (cleaved caspase-3 and cleaved caspase-9) in neurons were determined by western blotting. Data are shown as the mean ± standard deviation of three independent experiments. *p < 0.05, **p < 0.01, and ***p < 0.001 by one-way analysis of variance followed by Tukey’s post hoc test

To determine whether Mfn2 mediated the role of MOOs in astrocytic autophagy, we used siMfn2 to downregulate Mfn2 in the astrocytes (Fig. 6A). The PI3K/Akt/mTOR pathway modulates autophagy at the cellular level. We found that MOOs inhibited the activation of the PI3K/Akt/mTOR pathway in the LPS-stimulated astrocytes (Fig. 6B). However, Mfn2 downregulation reversed the regulatory role of MOOs in this pathway (Fig. 6B). Moreover, Mfn2 downregulation inhibited the formation of the autophagosomes and autolysosomes (Fig. 6C). Furthermore, Mfn2 downregulation decreased LC3 levels and increased p62 levels (Fig. 6D). Similar to 3-MA, Mfn2 downregulation led to an increase in cytokine levels (Fig. 7A), ROS accumulation (Fig. 7B, C), and mitochondrial damage (Fig. 7D) during MOO treatment. In the neurons co-cultured with the astrocytes, Mfn2 downregulation reduced their cell viability (Fig. 7E) and induced a mass of apoptotic neurons during MOO treatment (Fig. 7F, G).

### Mfn2 downregulation abrogated the role of MOOs in the depression-like behaviors of the hypertensive rats

**Fig. 8:**
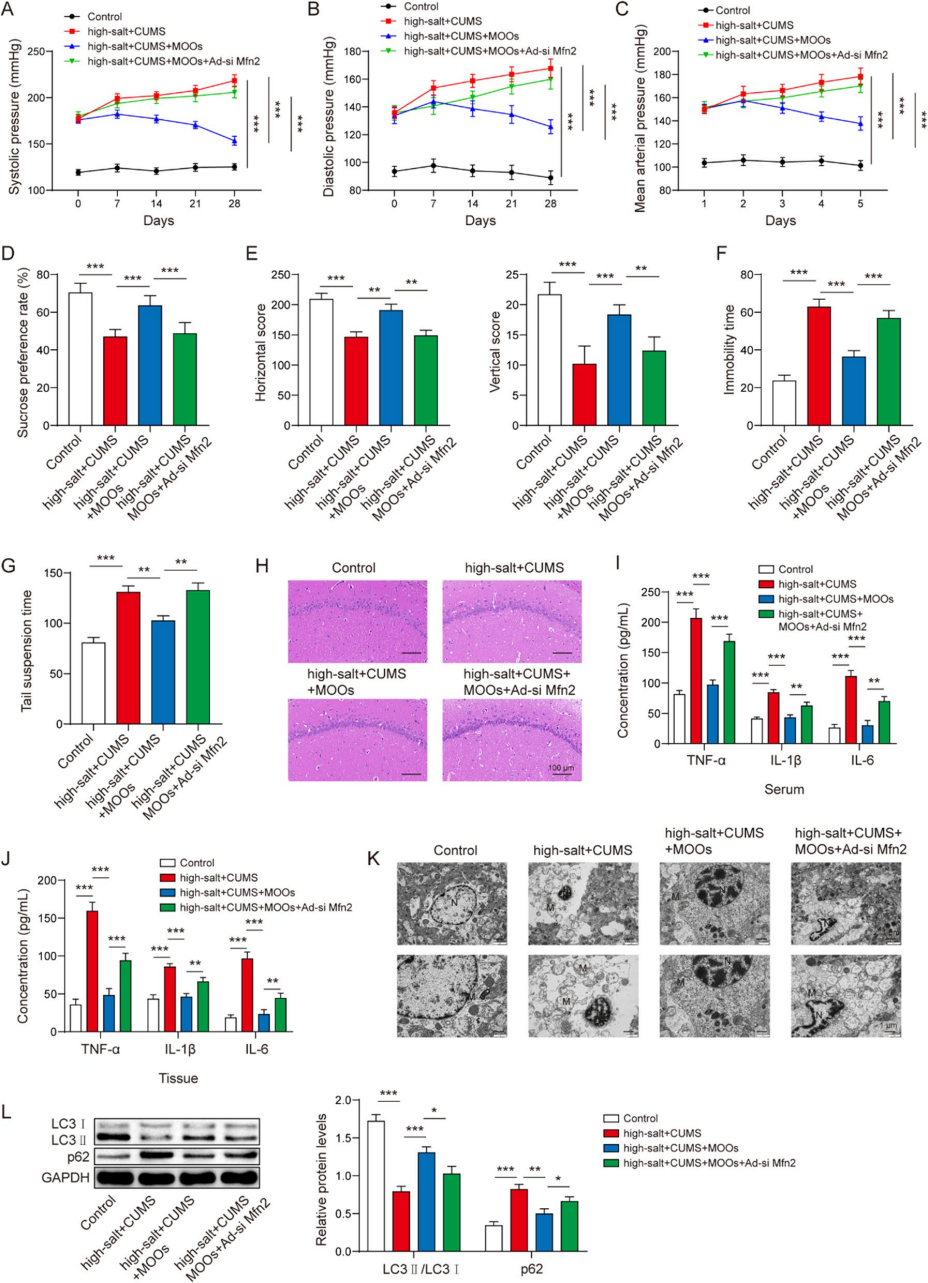
Mfn2 downregulation abrogated the effect of *Morinda officinalis* oligosaccharides on depression-like behaviors in hypertensive rats. The systolic pressure (**A**), diastolic pressure (**B**), and the mean arterial pressure (**C**) of the indicated rats. Sucrose preference test (**D**), open-field test (**E**), forced swimming test (**F**), and tail suspension test (**G**) of the indicated rats. **H** Histological changes in brain tissues were observed by hematoxylin and eosin staining. Tumor necrosis factor α, interleukin (IL)-1β, and IL-6 levels in serum (**I**) and brain tissues (**J**) were determined by enzyme-linked immunosorbent assay. **K** Mitochondrial morphology was observed using a transmission electron microscope. **L** Autophagy-related protein levels, including LC3 and p62, were determined by western blotting (*n* = 8 rats/group). Data are shown as the mean ± standard deviation. *p < 0.05, **p < 0.01, and ***p < 0.001 by one-way analysis of variance followed by Tukey’s post hoc test

We used adenovirus-packaged siMfn2 to downregulate Mfn2 expression in the depressive and hypertensive rats during MOO treatment. We showed that MOOs improved the hypertension symptoms in the depressive and hypertensive rats. Mfn2 downregulation induced an increase in the systolic pressure (Fig. 8A), diastolic pressure (Fig. 8B), and mean arterial pressure (Fig. 8C) during MOO treatment. Regarding the depression-like behaviors, Mfn2 downregulation decreased pleasure (Fig. 8D) and enthusiasm (Fig. 8E) and increased behavioral despair (Fig. 8F, G). Moreover, Mfn2 downregulation reversed the role of MOOs in histopathological changes (Fig. 8H), inflammation in the brain and serum (Fig. 8I, J), mitochondrial damage (Fig. 8K), and autophagy (Fig. 8L). Therefore, we determined that Mfn2 downregulation abrogated the role of MOOs in the depression-like behaviors of hypertensive rats.

## Discussion

**Fig. 9:**
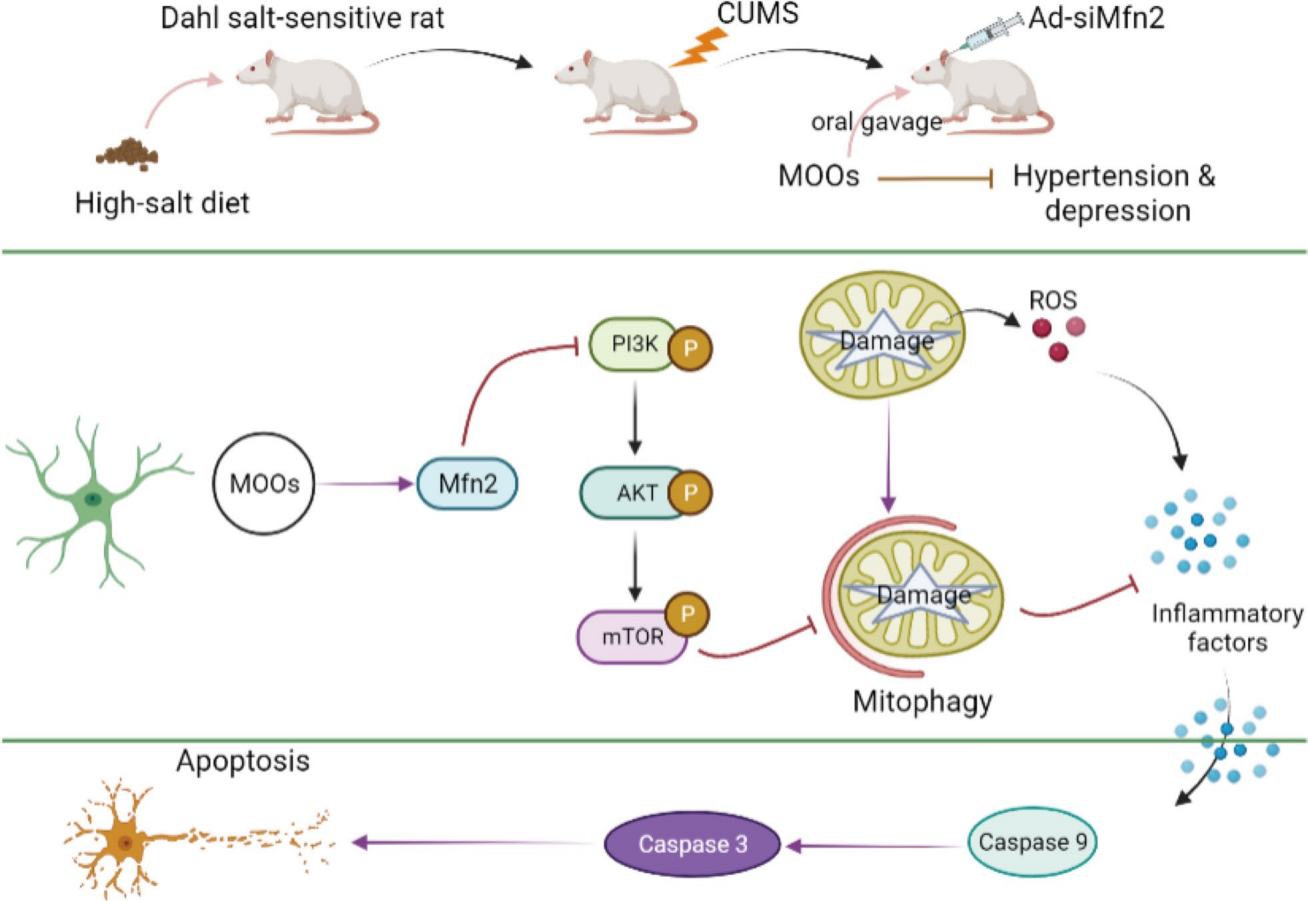
Schematic illustration of *Morinda officinalis* oligosaccharides’ functional role and potential mechanism in hypertension with depression

Generally, patients with hypertension suffer from depression [24]. In the present study, we showed that MOOs have anti-depression potential. However, whether MOOs can improve depression-like symptoms during hypertension remains unclear. We determined a novel therapeutic mechanism of MOOs in hypertension with depression (Fig. 9). First, we found that MOOs modulated astrocytic mitophagy to improve depression-like symptoms along with resisting hypertension. Then, we showed that Mfn2 expression played a core role in astrocytic mitophagy during MOO treatment. At the molecular level, MOOs upregulated Mfn2 expression to activate astrocytic mitophagy via the inactivation of the PI3K/Akt/mTOR pathway, thereby contributing to the removal of impaired mitochondria that caused oxidative stress and neurotoxicity in the astrocytes.

MOOs play anti-hypertension and anti-depression roles in vivo. We found that MOO treatment improved the depression-like behaviors along with the decrease in hypertension symptoms in the rats with CUMS. Hypertension may promote the occurrence and development of depression based on astrocytic dysfunction. We observed the decreased astrocytes and mitochondrial damage in brain tissues during hypertension which were aggravated by depression progression. Astrocytes are the largest population of glial cells. Astrocytes can release pro-inflammatory cytokines in response to stress, developing neuroinflammation and neurotoxicity in the CNS, which are the potential causes of depression [25–27]. In the pathological condition, impaired mitochondria lead to ATP dysfunction and ROS accumulation in astrocytes, ultimately resulting in astrocytic dysfunction [28]. Thus, hypertension causes mitochondrial damage in astrocytes to form the pathological basis of depression. The oral administration of MOOs can affect neuroinflammation and glia activation. Shi et al. used an ultra high-performance liquid chromatography–tandem mass spectrometry to detect the chemical components of *Morinda officinalis* root [29]. The chemical components were found in the brain of rats, suggesting the bioavailability of the components of Morinda officinalis root. The previous reports also indicate MOOs can be absorbed and then regulate NLRP3 inflammasomes, neuronal impairment ant other dysfunction associated with depression in the brain [20, 21, 30].

Intriguingly, we determined that MOOs played an anti-depressive role by targeting impaired astrocytes. During MOO treatment, we found increased astrocytes and more mitochondria with the complete structure in the hypertensive rats with depression-like symptoms. Existing evidence shows the anti-depression role of MOOs on the basis of NLRP3-mediated inflammation, cerebral 5-HT production, and microbiota-associated energy metabolism [19–22]. MOOs also affected the growth and survival of the astrocytes during depression, according to our findings. In this study, we provided a novel insight into the relationship between MOOs and impaired astrocytes showing that MOOs mitigated mitochondrial damage in the astrocytes to ameliorate neuroinflammation and depression-like behaviors during hypertension.

Importantly, we determined that MOOs possibly triggered mitophagy to remove the impaired mitochondria in the astrocytes. Mitophagy is a self-sustaining mechanism at the cellular level that maintains the quantity and quality of a mitochondrion [31]. Mitophagy is instrumental in the clearance of mtROS and mitochondrial DNA that impair normal glia in the pathophysiology of depression [32]. Mitophagy activation is an ideal therapeutic strategy for the management and prevention of depression during hypertension. In the LPS-induced astrocytes, we noticed that MOO treatment evoked mitophagy activation in astrocytes that contributed to the removal of ROS both in the cytoplasm and mitochondrion. Mitophagy-mediated mitochondrial quality control can ensure cell survival and cellular biofunction [33]. Astrocyte survival benefited from MOO-activated mitophagy. Moreover, we verified the mitophagy-mediated mechanism of MOOs in depression using 3-MA, an inhibitor of autophagy. Thus, we showed a new mechanism of MOOs in anti-depression. In hypertension with depression, MOOs activated mitophagy to protect the astrocytes from mitochondrial damage.

MOOs-activated mitophagy was mediated by Mfn2. Mfn2 is a mitochondrial fusion protein that participates in the regulation of mitochondrial fusion for mitochondrial maintenance. A dynamic balance between fusion and fission exists in the mitochondrion. Mfn2-mediated fusion forms mitochondrial, whereas Drp1-mediated fission causes mitochondrial fragments to be degraded by mitophagy [34]. A recent study found that the disruption of mitochondrial maintenance was characterized by differently expressed mitochondrial dynein [35]. Mfn2 can affect depression-like behaviors in vivo [36]. We found that Mfn2 was significantly decreased than other mitochondrial dyneins, including Drp1, Fis1, Mfn1, and OPA1. Possibly, Mfn2 downregulation disrupted the balance between the formation and degradation of mitochondria, ultimately resulting in mitochondrial damage in the astrocytes during hypertension with depression. Owing to MOO treatment, Mfn2 expression was increased in the astrocytes, promoting the normal operation of the dynamic balance between fusion and fission. Notably, Mfn2 induced the inactivation of the PI3K/Akt/mTOR pathway, which is a crucial regulator of autophagy at the cellular level [37]. Mfn2 mediated the anti-depressive role of MOOs with the PI3K/Akt/mTOR pathway during hypertension with depression. Collectively, we determined that Mfn2 was the therapeutic target of MOOs to modulate mitophagy activation in the astrocytes during hypertension with depression.

Although we determined the alteration of inflammatory cytokines and histology in brain tissues, we failed to investigate these parameters in the specific brain regions, which is the limitation of our investigation. We aim to show the changes in these parameters in depression-related brain regions such as the hippocampus and the prefrontal cortex in follow-up studies. In addition, we observed Mfn2 expression in LPS-stimulated cells treated with MOOs at 1.25 mg/mL was decreased as compared to LPS group. However, Mfn2 was increased because of MOOs treatment at 2.5 and 5 mg/mL. Possibly, 1.25 mg/mL MOOs was not enough to reversed the inhibitory role of LPS in Mfn2 expression. Further, MOOs at low concentration might contributed to the LPS-decreased Mfn2 expression. There is a potential threshold of MOOs to reverse the interaction between MOOs and LPS in astrocytes, which can be explored in follow-up study.

To summarize, we provided a novel anti-depressive mechanism of MOOs on the basis of astrocytic mitophagy. We found MOOs upregulated Mfn2 expression to activate the PI3K/Akt/mTOR pathway-mediated mitophagy, which removing impaired mitochondria in astrocytes during hypertension with depression. The intriguing connection between MOOs and astrocytic mitophagy will contribute to the management and treatment of various mental disorders occurring during hypertension. Furthermore, we determined the significant role of Mfn2 in mitophagy activation. Hence, Mfn2 is the potential therapeutic target in hypertension with depression.

## Data Availability

The datasets used or analyzed during the current study are available from the corresponding author on reasonable request.

## Abbreviations

MOOs: *Morinda officinalis* oligosaccharides
LPS: Lipopolysaccharide
3-MA: 3-Methyladenine
TNF-α: Tumor necrosis factor-α
IL-6: Interleukin 6
IL-1β: Interleukin 1β
CNS: Central nervous system
ROS: Reactive oxygen species
Mfn2: Mitofusion 2
Cyt-C: Cytochrome C
CUMS: Chronic unpredictable mild stress
ELISA: Enzyme-linked immunosorbent assay
qPCR: Quantitative polymerase chain reaction
CCK-8: Cell Counting Kit-8
MOI: Multiplicity of infection
PVDF: Polyvinylidene difluoride
